# Recombinant Plants Provide a New Approach to the Production of Bacterial Polysaccharide for Vaccines

**DOI:** 10.1371/journal.pone.0088144

**Published:** 2014-02-03

**Authors:** Claire M. Smith, Stephen C. Fry, Kevin C. Gough, Alexandra J. F. Patel, Sarah Glenn, Marie Goldrick, Ian S. Roberts, Garry C. Whitelam, Peter W. Andrew

**Affiliations:** 1 Department of Infection, Immunity and Inflammation, University of Leicester, Leicester, Leicestershire, United Kingdom; 2 The Edinburgh Cell Wall Group, Institute of Molecular Plant Sciences, School of Biological Sciences, University of Edinburgh, Edinburgh, United Kingdom; 3 School of Veterinary Medicine and Science, University of Nottingham, Sutton Bonington Campus, Leicestershire, United Kingdom; 4 Faculty of Life Sciences, University of Manchester, Manchester, United Kingdom; 5 Department of Biology, University of Leicester, Leicester, Leicestershire, United Kingdom; Instituto Butantan, Brazil

## Abstract

Bacterial polysaccharides have numerous clinical or industrial uses. Recombinant plants could offer the possibility of producing bacterial polysaccharides on a large scale and free of contaminating bacterial toxins and antigens. We investigated the feasibility of this proposal by cloning and expressing the gene for the type 3 synthase (*cps3S*) of *Streptococcus pneumoniae* in *Nicotinia tabacum*, using the pCambia2301 vector and *Agrobacterium tumefaciens*-mediated gene transfer. *In planta* the recombinant synthase polymerised plant-derived UDP-glucose and UDP-glucuronic acid to form type 3 polysaccharide. Expression of the *cps3S* gene was detected by RT-PCR and production of the pneumococcal polysaccharide was detected in tobacco leaf extracts by double immunodiffusion, Western blotting and high-voltage paper electrophoresis. Because it is used a component of anti-pneumococcal vaccines, the immunogenicity of the plant-derived type 3 polysaccharide was tested. Mice immunised with extracts from recombinant plants were protected from challenge with a lethal dose of pneumococci in a model of pneumonia and the immunised mice had significantly elevated levels of serum anti-pneumococcal polysaccharide antibodies. This study provides the proof of the principle that bacterial polysaccharide can be successfully synthesised in plants and that these recombinant polysaccharides could be used as vaccines to protect against life-threatening infections.

## Introduction

Polysaccharide encapsulated bacteria are major causes of disease and death in humans and animals. For example, diseases caused by *Streptococcus pneumoniae* (the pneumococcus), *Neisseria meningitidis* and *Haemophilius influenzae* are responsible for more than two million deaths every year, the majority children under the age of five [1], [2] [1], [2]. *Streptococcus pneumoniae* alone is responsible for more than 50 percent of invasive disease worldwide. Despite the extensive use of pneumococcal vaccines, incidences of disease caused by *S. pneumoniae* remain high, mainly due to serotypes not included in the vaccine [3]. Current anti-pneumococcal vaccines are composed of capsular polysaccharide alone or conjugated to protein. Whatever the formulation, pneumococcal vaccine design has to deal with the facts that there are over 90 different capsular and the serotype distribution varies with time and geography. However, for reasons of economics and biology the current vaccines are limited in coverage (23 in the polysaccharide-only vaccine and 13 in the new version of the conjugate) to the most dominant serotypes in Europe and North America. Ideally multiple versions of these vaccines are required and they would be regularly reformulated to offer maximum protection. Cost of polysaccharide production then becomes a concern. One of the challenges for pneumococcal vaccine production is to manufacture bacterial polysaccharide on a large-scale, without need for purification procedures to remove contaminating toxins and pyrogens. Currently the preparation of polysaccharides requires expensive fermentation equipment, microbiological containment and high levels of quality control to prevent contamination. Plants offer a solution because they synthesise a large number of high molecular weight polysaccharides, they have many of the sugar precursors of bacterial capsular polysaccharide readily available and plants have compartmentalised metabolic pathways and transport processes that could facilitate polysaccharide extraction [4]. However, until now heterologous antigen production in plants has been limited to the production of proteins [5], [6], [7]. Here we report that plants can be engineered to synthesise bacterial polysaccharides and these polysaccharides provide protective immunity. We demonstrated this principle using the serotype 3 capsular polysaccharide of *S. pneumoniae*, a serotype that is frequently isolated from disease cases. The type 3 polysaccharide is composed of repeating d-glucose (Glc) and d-glucuronic acid (GlcA) units, as (1→4)-β-d-Glc*p*-(1→3)-β-d-Glc*p*A-(1→4) [8], [9] The precursors, UDP-glucose (UDP-Glc) and UDP-glucuronic acid (UDP-GlcA), are polymerised by a type 3 synthase (Cps3S) [8], [9].

## Results

### The Pneumococcal Type 3 Capsule Synthase Gene was Cloned into *Nicotinia tabacum* by *Agrobacterium*-mediated Gene Transfer

The pneumococcal *cps3S* gene {Dillard, 1995 #888} was amplified from genomic DNA of the pneumococcal type 3 strain WU2 using primers CPSFOR and CPSREV and cloned with PR1b signal sequence (which was used to direct secretion of the transgene to the apoplast) into the *Agrobacterium* binary vector pCambia 2301, to give pCMS4. This placed *cps3S* under the control of duplicated constitutive cauliflower mosaic virus promoters, CaMV35S and also enabled selection of transformed plants with kanamycin. Nucleotide sequence analysis of the cloned *cps3S* in pCMS4 showed 100 % identity with the published sequence [8].

**Figure 1:**
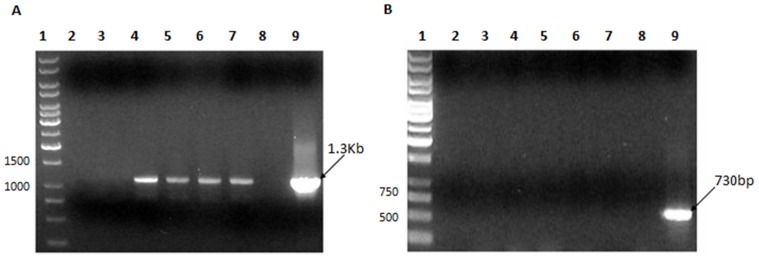
Detection of the *cps3S* gene in transformed tobacco plants. **A.** DNA was used as a template for PCR (Lanes 2, 3: wild type plants; Lanes 4 – 7: transformed plants.) using *cps3S*-specific primers. PCR products were analysed by agarose gel electrophoresis. The results show the presence of the *cps3S* gene in the transformed plants (Lanes 4 - 7) but not the wild type plants. The PCR reaction in Lane 9 contained purified plasmid DNA containing *cps3S* (pCMS4) as a positive control and Lane 8 contained no template DNA. Molecular sizes are indicated. **B**. PCR showing the absence of *Agrobacterium* DNA contaminating DNA preparations from wild type (Lanes 2, 3) and transformed (Lanes 4 - 7) tobacco plants. PCR was done with *Agrobacterium*-specific primers. The results show that there was no *Agrobacterium* DNA present in the transgenic plant samples. The PCR reaction in Lane 9 contained *Agrobacterium* DNA as a positive control and shows the expected 730bp band and Lane 8 contained no template. Molecular sizes are indicated. DNA was extracted from the same six *N. tabacum* plants for the PCRs shown in A and B.

**Figure 2:**
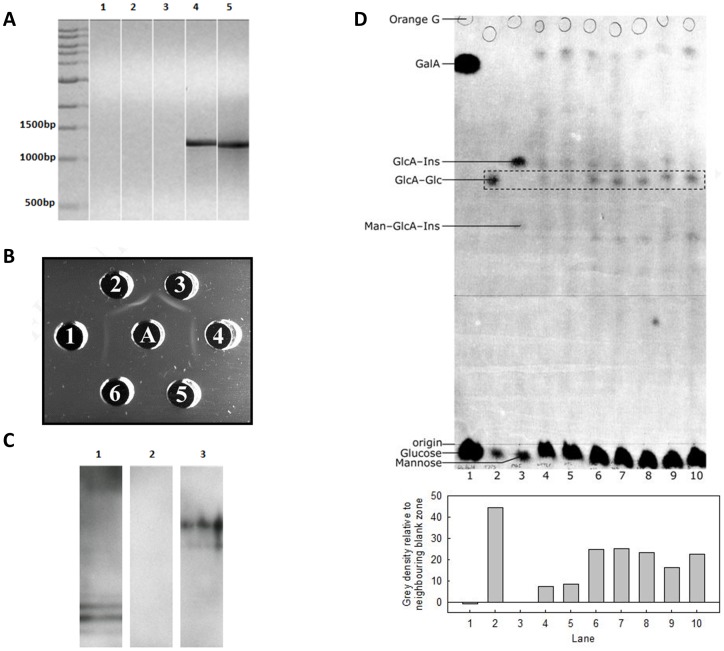
*In planta* expression of the *cps3S* gene and formation of serotype 3 polysaccharide. **A.** Reverse transcriptase PCR to detect *cps3S* mRNA in transgenic tobacco plants. RNA was extracted from a wildtype (Lanes 1 and 3) and a transgenic *N. tabacum* containing *cps3S* (Lanes 2 and 4). PCR products, using *cps3S* specific primers were analysed by agarose gel electrophoresis. Lanes 1 and 2 showed the absence of *cps3S* DNA in the RNA. RT-PCR on the same samples showed the presence of *cps3S* mRNA in the transgenic plant (Lane 4) but not in the wildtype (Lane 3). Lane 5 PCR of pCMS4 containing *cps3S*, done as before. The 1.3 kb amplicon in Lanes 4 and 5 shows a full-length transcript of *cps3S* is expressed in the transgenic plant. **B.** Double immunodouble diffusion. Well 1∶10 µg purified serotype 3 polysaccharide; Wells 2-4: extract from tobacco plants shown to express *cps3S*: Wells 5 and 6: extract from a wildtype tobacco plant. Well A: type 3 polysaccharide specific antiserum. The preciptin lines identify the presence of type 3 polysaccharide. **C.** Western blotting using type 3 polysaccharide specific antiserum. Lane 1: purified type 3 polysaccharide; Lane 2: wildtype plant extract; Lane 3: transgenic plant extract. **D**. High-voltage paper electrophoresis of tobacco leaf acid hydrolysates. Lanes 1-3: 25 µg of each marker, (Lane 1) galacturonic acid (GalA) and glucose, (Lane 2) glucose and β-d-glucuronosyl-(1→4)-d-glucose (GlcA–Glc) (partial hydrolysate of 10 µg type 3 pneumococcal polysaccharide) and (Lane 3) 10 µg of a mixture of mannose, α-d-glucuronosyl-(1→2)-*myo*-inositol (GlcA–Ins) and a trace of α-d-mannosyl-(1→4)-α-d-glucuronosyl-(1→2)-*myo*-inositol (Man–GlcA–Ins). Lanes 4–10: hydrolysate of polysaccharides cold-acid-extracted from 32 mg fresh weight of wildtype (Lanes 4 and 5) or transgenic (Lanes 6–10) tobacco leaves. Each lane also contains a trace of Orange G (coloured internal marker). All lanes show similar levels of staining for neutral sugars (co-migrating with glucose, near the origin). The samples were electrophoresed at pH 6.5, at 3.0 kV for 60 min (anode at top) and stained with AgNO_3_. Spots of the disaccharide, GlcA–Glc, diagnostic of type 3 pneumococcal polysaccharide, are highlighted by the dashed box; these spots were quantified for grey density in PhotoShop (see histogram).

*Nicotiana tabacum* was transformed with pCMS4 by *A. tumefaciens*-mediated gene transfer. A T1 generation was grown from the seeds of six plants and PCR showed that four plants contained the 1.3kb *cps3S* gene (Figure 1A). PCR also confirmed the absence of contaminating *Agrobacterium* DNA (Figure 1B). RT-PCR, with *cps3S*-specific primers, showed that the transgene was expressed in the transgenic plants (Figure 2). No amplicon was generated by direct PCR amplification of RNA extracts, confirming the absence of contaminating *cps3S* DNA (Figure 2A). No amplicon was generated by RT-PCR of untransformed plants (Figure 2A lane 3). A second generation of plants were grown from the seeds of these plants and PCR confirmed stable transgene expression (Figure S1). All subsequent assays were done with second generation (T2) plant material.

### Pneumococcal Type 3 Polysaccharide was Detected in the Leaves of Transformed Plants

Double immunodiffusion showed that type 3 antibody-antigen complexes were seen (Figure 2B) between wells which contain purified type 3 pneumococcal polysaccharide, sonicated plant cell extract from transgenic plants (wells 1-4) and type 3 polysaccharide specific antiserum (well A). This was not seen in wells containing extract from a wildtype tobacco plants (wells 5 and 6). Western blotting of transgenic and wildtype plant extracts using type 3 polysaccharide specific antiserum also showed the presence of type 3 polysaccahride in transgenic plant extracts only (Figure 2C). High-voltage paper electrophoresis of hot-acid hydrolysates of cold-acid-extractable tobacco leaf polysaccharides confirmed these findings (Figure 2D). Acid hydrolysis of polysaccharides from transgenic leaves produced a relatively hot-acid-resistant, singly-ionised disaccharide with the same mobility as the β-d-Glc*p*A-(1→4)-d-Glc seen following acid hydrolysis of pneumococcal type 3 polysaccharide. This disaccharide was barely detectable in wild type non-transformed plants (Figure 2D).

### Immunisation with Transgenic Plant Extracts Protected Mice from Pneumococcal Disease

**Figure 3:**
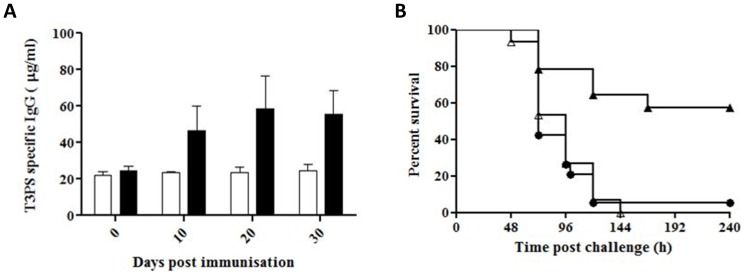
Immunogenicity and protective efficacy of serotype 3 pneumococcal polysaccharide produced *in planta*. **A.** Concentration of serotype 3 polysaccharide-specific IgG in serum of mice immunised with extracts from tobacco plants expressing *cps3S* (black bars) or wildtype plant (white bars); n  =  5. **B.** Survival of mice challenged with virulent type 3 pneumococci 230 days after the final immunisation with transgenic plant extract (closed triangles), wildtype extracts (open triangles) or sham-immunised mice (closed circles). Mice alive at 240h post-infection were considered to have survived the infection.

To test the immunogenicity of the plant-derived type 3 polysaccharide, mice were immunised with three doses of apoplast extracts from transgenic or wildtype plants. Sera were collected on the day before each immunisation and ten days after the final dose, and anti-type 3 polysaccharide IgG was determined by ELISA. Significantly more (P<0.05) specific anti-type 3 antibody was detected after a single dose of the transgenic leaf extract, with a further increase (P<0.05) after a second dose (Figure 3A), whereas antibody levels remained unchanged in those given wildtype extracts (P>0.05).

Mice were challenged intranasally with the serotype 3 *S. pneumoniae* strain HB565 230 days after the final immunisation. Mice immunised with transgenic plant extract survived significantly longer (P<0.001) than those given wildtype extracts (mean survival: 181±72h and 90±23h for transgenic and wildtype, respectively). Mice immunised with the wildtype extract did not survive longer (P>0.05) than sham-immunised mice (91±38h). None of the fifteen animals given wildtype extract were alive ten days after the challenge, whereas eight of the fourteen immunised with transgenic extract survived (Figure 3B).

## Discussion

This study has shown that bacterial polysaccharide vaccine antigens can be synthesised in plants and that simple extracts of these plants are immunogenic and protect against an otherwise lethal infection. Transgenic plants are recognised as good expression systems for proteins but the synthesis of bacterial polysaccharide in plants has not been demonstrated before.

We chose the production of the type 3 polysaccharide of *S. pneumoniae* for the reason that it is a relatively simple carbohydrate, being composed of repeating d-glucose (Glc) and d-glucuronic acid (GlcA) organised as (1→4)-β-d-Glc*p*-(1→3)-β-d-Glc*p*A-(1→4) units [8], [9]. The precursors, UDP-glucose (UDP-Glc) and UDP-glucuronic acid (UDP-GlcA) are naturally synthesised by plants, which transport them into the endomembrane system as substrates for cell wall polysaccharide synthesis [4], [10]. Polymerisation of these substrates into type 3 polysaccharide requires the enzyme, type 3 synthase (Cps3S). Therefore, we cloned the pneumococcal *cps3S* gene into *N. tabacum* using pCambia 2301. This strategy not only placed *cps3S* under the control of duplicated constitutive cauliflower mosaic virus promoters, CaMV35S, but it also enabled selection of transformed plants with kanamycin. Growth of a second generation of kanamycin-resistant plants confirmed stable transgene expression. Although not the primary purpose of the study, we did a limited investigation of how to extract the pneumococcal polysaccharide from plant tissue. The method that yielded the highest concentration of pneumococcal polysaccharide was to grind the plant tissue under liquid nitrogen, suspend the tissue in water and lyse the cells by sonication. Despite cloning the signal sequence PR1b we detected no type 3 polysaccharide in its destination, the apoplastic fluid. This implied that PR1b was not functioning correctly. Previous studies replaced the start codon of the transgene with PR1b [3], however, we maintained the start codon and cloned an in-frame sequence of *cps3S*. This may have led to a reduction in PR1b activity and improving this may increase the yield of type 3 polysaccharide. Another method to increase yield is to use root tissues, since the continuously growing primary cell wall may contain higher concentrations of the UDP-precursors. In this study we focussed on leaf tissue as we were working with parent and F1 generations and removal of the roots may have restricted growth of the plant and seed development. Leaf tissue was also much easier to obtain. For these reasons, the levels of polysaccharide extracted from plant tissue may not have been optimal.

Having shown the principle of *in planta* synthesis using the linear type 3 polysaccharide, the next challenge is the production of more complex, branched, bacterial polysaccharides. All the genes involved in pneumococcal capsular polysaccharide synthesis are closely linked on the bacterial chromosome, arranged within a single locus (a “type specific” cassette). Therefore, it is possible that the introduction of whole cassettes could lead to the synthesis of sugar precursors not naturally occurring in the plant and their assembly into more complex polysaccharides. Furthermore, effective signal or transport peptides should allow easier extraction by compartmentalising different polysaccharides.

Because anti-polysaccharide antibodies are protective against several bacterial pathogens of humans and animals there is great interest in polysaccharides as vaccines. However, some of the problems with these vaccines are illustrated by vaccines against *S. pneumoniae*. The current pneumococcal vaccine contains twenty-three polysaccharide serotypes but protection is serotype-specific and some are not immunogenic in children. The vaccine was formulated on the prevalence of serotypes in North America and Europe, but elsewhere the coverage can be considerably less [11]. In addition to protection being serotype-specific, polysaccharide immunogenicity also varies with serotype and age [12]. Furthermore, temporal variation occurs in the serotypes isolated from adults and children [13]. Thus it has been suggested that different vaccine formulations should be manufactured for differing situations [14], but unless low-cost solutions are found it will not become a reality. Polysaccharide vaccines can be expensive, which restrains their use in developing countries. Production of polysaccharide vaccines in plants can introduce economies of scale that can drive down the production costs. The alternative, of using microorganisms as the vaccine production system, requires expensive fermentation equipment and high levels of quality control to prevent contamination. In contrast, the use of plants for vaccine production offers an achievable solution, opening the possibility of local production, which increases the likelihood of adoption of the vaccine [15].

Efforts to improve the poor immunogenicity of polysaccharide vaccines in the young are focused on the development of polysaccharides covalently linked to protein, but these make difficulties with serotype coverage worse. When the US FDA licensed a 7-valent anti-pneumococcal conjugate vaccine (Prevnar) the serotypes covered by the vaccine caused 90% of disease in North America and Europe, but less than 70% in Asia [15]. This emphasises the desirability of a ‘tailor-made’ vaccine. However, formulations of conjugates for a particular country or for childhood and for adult vaccination programmes are even less likely than pure polysaccharide formulations, unless cheaper production methods can be found. Conjugate vaccines are very expensive and the pneumococcal conjugates will be more expensive than any other current vaccine. Even with tiered pricing the price of these vaccines is a real concern for their uptake [16]. The ability to produce polysaccharide vaccines in plants, on a large scale, could lead to a ready availability of polysaccharides for protein conjugate vaccine production. There is, however, a more exciting possibility, namely to exploit the plant’s glycosylation machinery to glycosylate heterologous proteins with heterologous polysaccharide to make conjugate vaccines *in planta*. Plants making heterologous immunogen represent an innovative technology for the development of childhood vaccines. The longer-term objective of this research is to synthesise polysaccharide-protein conjugates in plants.

*In planta* synthesis of bacterial polysaccharide and conjugates offers an innovative contribution to vaccinology. Synthesising heterologous polysaccharides in plants represents the first proof of concept step in this process. These experiments have yielded exciting data even though no attempt was made to optimise gene expression, polysaccharide purification or immunising protocol. These are developmental issues for the future now that the concept that bacterial polysaccharide can be synthesised in plants has been proven.

## Materials and Methods

### Construction of the Plant Expression Vector

**Figure 4:**
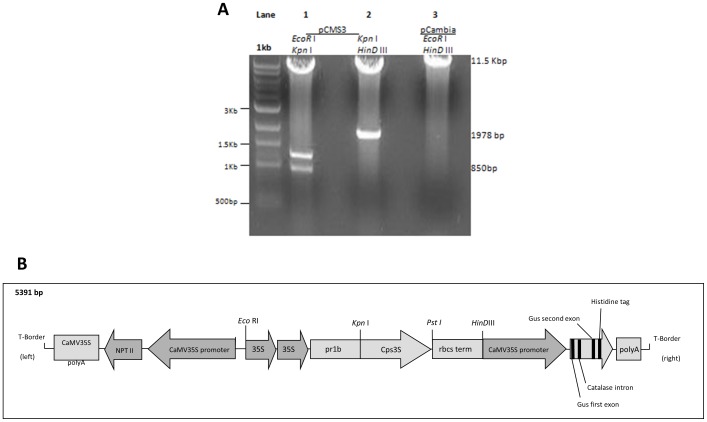
Construction of the pCMS4 vector. **A.** The digested products of three separate restriction digests of pCMS3 and pCambia 2301. Lane 1 shows the expected 3 fragments of pCMS3 when digested with EcoR I and Kpn I. Lane 2 shows the expected 2 fragments produced from the digestion of pCMS3 with Kpn I and HinD III. Lane 3 shows the expected band of 11.5 Kbp when pCambia 2301 was digested with EcoR I and HinD III. The DNA fragments of 864bp (Lane 1) and 1978bp (Lane 2), indicated by the arrows, were used to reconstruct the pCMS3 T-DNA fragment containing cps3S and these were ligated to the 11.5 Kb pCambia fragment (Lane 3). This plasmid was termed pCMS4. Undigested DNA is not shown. A 1 Kb ladder (New England Biolabs) was used as a molecular size marker. **B.** The expression vector pCHF2. A CaMV35S promoter, rbcS terminator region, and a PR1B signal sequence were cloned into the T-DNA region of the pPZP222 vector [17]. The unique restriction endonuclease sites are also shown.

The sequence of the type 3 pneumococcal capsular polysaccharide biosynthesis cassette was obtained from GenBank (www.ncbi.nlm.nih.gov), accession number U15171. A 1.3 kb DNA fragment containing the *cps3S* gene was obtained by PCR from the genomic DNA of *S. pneumoniae* serotype 3 (WU2) using the oligonucleotide primers sense (CPSFOR) 5′-CTG GTA↓CCC ATG TAT ACA TTT ATT TTA ATG TTG TTG G-3′ corresponding to 2227bp - 2254bp with a *KpnI* restriction site inserted at the 5′ end; anti-sense (CPSREV) 5′- TCA TCA CTC TGT TAA ATT CCT AGT TCC -3′, corresponding to 3454bp - 3479bp of the cassette. The PCR amplification was performed in a total volume of 50 µl. using 2 µg genomic template under the following conditions: 94°C for 4 minutes and then a cycling procedure comprising denaturation at 94°C for 45 seconds, annealing at 58°C for 1 minute, and extension at 72°C for 1 minute 30 seconds, which was repeated either 10 or 30 times, and a final extension at 72°C for 10 minutes. Amplified DNA resulting from PCR were purified from the PCR reagents using the QIAquick PCR purification kit (Qiagen). The amplified fragment was inserted into the multiple cloning site of pCR4-TOPO (Invitrogen, Carlsbad, CA, USA). The *Agrobacterium* binary vector pPZP221 (GenBank U10463) [17] has been previously engineered to produce vector pCHF2 (Figure 4) containing the constitutively expressed cauliflower mosaic virus (CaMV) 35S promoter and a *rbcS* terminator (C. Fankhauser, personal communication). Here, synthetic primers homologous to the PR1b signal peptide sequence [18] were annealed, phosphorylated and inserted into pCHF2 using *Sac*I and *Kpn*I cutting sites situated between the CaMV 35S promoter and the *rbcS* terminator. The *cps*3S gene was removed from the pCR4-TOPO vector using *Kpn*I and *Pst*I and inserted between the PR1b signal sequence and the *rbcS* terminator sequence, to form the clone pCMS3. The entire expression cassette was then excised from pCMS3 with *EcoR*I and *HinD*III and ligated into the binary plant vector pCAMBIA2301 (GenBank accession number AF234316) (containing a kanamycin-resistance gene and *gus*) to give the resulting plasmid, pCMS4.

### Transformation of Plants

pCMS4 was introduced into *Agrobacterium tumefaciens* strain GV3101 directly by the heat shock method, using 0.5 µl (1 µg) pCMS4 and 0.1 ml of frozen CaCl_2_-competent *A. tumefaciens* cells. Cells were thawed at 37°C for 5 minutes, re-suspended in 1 ml of YEP broth (10 g/L Yeast Extract, 10 g/L Peptone, 5 g/L NaCl, pH 7.0) and incubated at 28°C for 2 hours with gentle shaking. Cells were harvested by centrifugation at 600 *g* for 10 minutes, the pellet re-suspended in 0.1 ml YEP and spread onto YEP agar containing 100 µg/ml kanamycin and 100 µg/ml rifampicin and incubated overnight at 28°C. Subsequently, *A. tumefaciens* carrying pCMS4 was used to transform tobacco *(Nicotiana tabacum cv* SR1) leaf discs, as described previously [19]. Leaf discs were then transferred to Murashige-Skoog (MS) agar containing 3% (w/v) sucrose, the plate was sealed with parafilm and incubated in the dark at room temperature for 2 days. Discs were then transferred to selective medium (MS agar containing 3% (w/v) sucrose, 250 µg/ml cefotaxime (to kill the *A. tumefacien*s on the surface of explants), 100 µg/ml kanamycin, 100 µg/ml ampicillin, 100 µg/ml naphthalene acetic acid (NAA) and 1 mg/ml 6-benzylaminopurine) to select transgenic progeny. Ten days post-infection, those discs showing shoot formation were transferred to fresh selective medium and re-incubated for approximately 1-2 weeks. Larger shoots were transferred to powder rounds containing the same medium and incubated for a further 3 weeks, or until roots started to form. After eight weeks, kanamycin-resistant shoot regenerants were removed to a rooting medium containing 100 µg/ml NAA and 25 µg/ml kanamycin. Rooted plantlets were transferred to soil, self-pollinated and the seeds stored desiccated at 4°C.

### Detection of *cps3S* Gene Expression in Transgenic Plants

Total RNA was isolated from 100 mg of leaf tissue using a RNA isolation kit (RNeasy Plant Mini Kit; Qiagen, Surrey, UK) and used in the production of first strand cDNA using a cDNA synthesis kit (RNase H^−^ reverse transcriptase kit; Invitrogen) and a random hexa-nucleotide primer. PCR was then performed using the CPSFOR and CPSREV primers as described above, RNA extracts without prior reverse transcriptase treatment were used as a control to indicate the presence of *cps3S* specific DNA. RNA extracted from wild-type tobacco leaves also was used as negative control. To confirm the absence of contaminating *Agrobacterium* DNA PCR was done with the primers VCF (5′-ATC ATT TGT AGC GAC T-3′) and VCR (5′-AGC TCA AAC CTG CTT C-3′), designed to amplify a 730bp region of the *virC* gene of Agrobacterial Ti and Ri plasmids [20].

### Preparation of Leaf Extracts

Leaves were collected from tobacco plants, tissue ground under liquid nitrogen and nanopure water added to give 0.5 g/ml plant tissue. The cells were lysed by sonication: 6×30 second sonications at an amplitude of 50 microns with 30 second rests in between sonications. The cell lysate then was centrifuged for 5 minutes at 10,000 *g* and the supernatant divided into 1 ml volumes, lyophilised and stored at 4°C.

### Extraction of type 3 Polysaccharide from Apoplastic Fluid

A modification of the method described by Fry and co-workers was used [21]. Leaf material (1 g) was added to 50 ml of 50 mM CaCl_2_ and vacuum-infiltrated for a period of 30 minutes. The leaves were removed and dried gently on a paper towel before being transferred to the barrel of a 25 ml syringe with the plunger removed. This was placed in a 50 ml Falcon tube and the assembly centrifuged at 800 *g* at 10°C for 10 minutes. The aqueous extract was stored at 4°C until required.

### Double Immunodiffusion

A modification Ouchterlony’s method was used [22]. 0.2% (w/v) Ouchterlony agarose in barbitone buffer (1.84 g/l diethylbarbituric acid, 10.3 g/l sodium diethylbarbiturate, pH 8.6) was used to coat microscope slides. These were left to dry for 1 hour, and then overlaid with 4.5 ml 1% (w/v) agarose in barbitone buffer. Once set, 4 mm holes were cut and 20 µl of sample was placed in the outer holes. Type 3 polysaccharide from *S. pneumoniae* (ATCC), diluted in 20 µl of untransformed plant extract, was used as a positive control. The central hole contained 20 µl neat rabbit anti-type 3 polysaccharide antiserum (Statens Serum Institute, Copenhagen, Denmark). The slides were incubated at 4°C in a humidity box for 1-2 weeks. Precipitin lines were observed and photographed in indirect light. Recombinant pneumococcal polysaccharides were estimated by comparison with the intensity of the precipitin lines of the positive controls.

### High-Voltage Paper Electrophoresis (HVPE)

Leaf material was harvested, washed, cut into pieces and ground into a fine powder under liquid nitrogen. Samples were stored at –20°C until required. To 10 g fresh weight was added 50 ml 5% (v/v) formic acid and the suspension was incubated with gentle shaking for 2 days at room temperature. This procedure is expected to extract the capsular polysaccharide, but only a small proportion of the leaf cell-wall polysaccharides and starch. The homogenate was filtered through Miracloth and rinsed with 25 ml water and then the combined filtrate was adjusted to pH 4.0 with pyridine. Co-extracted proteins were denatured at 100°C for 60 min, then cooled and pelleted by centrifugation at 1700×*g* for 15 min; the supernatant was freeze-dried. The dried material was washed exhaustively at room temperature in several changes of 82.6% (v/v) ethanol, which dissolves low-MW sugars. The remaining insoluble, polysaccharide-rich material was then air-dried, incubated at 90°C for 30 min in 22.6 ml water and cooled; trifluoroacetic acid (TFA) was then added to a final concentration of 0.36 M, which solubilised the polysaccharide.

For partial hydrolysis of the polysaccharide to yield the relatively acid-resistant, diagnostic dimer (aldobiouronic acid; GlcA–Glc) a portion of the solution was hydrolysed in 2 M TFA at 120°C for 30 min [conditions optimised in preliminary runs with authentic type 3 polysaccharide; data not shown], then dried *in vacuo*. The hydrolysis products were redissolved in water containing a trace of Orange G (internal anionic marker), and a volume (equivalent to 32 mg fresh weight of leaf) was spotted on to Whatman 3 MM paper. The samples were subjected to HVPE at pH 6.5, at 3.0 kV for 60 min [23] and then stained with silver nitrate [21] to reveal sugars. External markers, run on the same sheet, included hydrolysates of (i) purified Type 3 polysaccharide (yielding glucose plus GlcA–Glc), and (ii) the trimer α-d-mannosyl-(1→4)-α-d-glucuronosyl-(1→2)-*myo*-inositol [24] (which yields a comparable dimer, α-d-glucuronosyl-(1→2)-*myo*-inositol, plus mannose). Other markers were commercial glucose and galacturonic acid. After staining with silver nitrate [21], electrophoretograms were scanned and relevant spots were quantified for grey density in PhotoShop, as described in Supplementary Figure 1 of Parsons *et al* [25].

### Immunisation and Challenge

Nine-week-old MF1 female mice (HarlanOlac, Bicester, UK) were given three doses of control plant extract or plant extract containing 2 µg plant-derived pneumococcal polysaccharide per mouse (as estimated by the Ouchterlony method) in 67 µl PBS and 33 µl Imject alum adjuvant (Pierce, Rockford, IL, USA). Mice were immunised intraperitoneally on days 0, 10, 20 and 30. Sham-immunised mice received alum adjuvant containing an irrelevant immunogen (KLH) using the same schedule. Serum samples were obtained by tail bleeding the day before each immunisation. Mice were challenged intraperitoneally with 2.8×10^6^ cfu serotype 3 pneumococci on Day 260. The health status of animals was monitored, according to the scheme of Morton *et al* [26].These experiments were done under a project licence from the UK Home Office.

### ELISA

Maxisorb ELISA wells (Gibco BRL, Nunc products) were coated with 2 µg/ml purified type 3 pneumococcal polysaccharide (ATCC) in coating buffer (50 nM NaHCO_3_ pH 9.6, 0.02% (w/v) NaN_3_) for 16 h at 22°C. After rinsing with PBS, the wells were blocked with PBS + 5% (w/v) dried milk at 37°C for 1 h and washed three times with washing buffer (50 mM TrisHCl pH 7.5, 150 mM NaCl, 0.05% (v/v) Tween20). Mouse sera were diluted 1∶100 in blocking buffer and 100 µl added to the wells and incubated, shaking, for 2 hours at 37°C. The plates were washed three times as before and bound antibodies were detected using alkaline phosphatase-conjugated goat anti-mouse IgG secondary antibody (Fc specific, Sigma; diluted 1∶5000), and 1 mg/ml p-nitrophenyl phosphate (Sigma) dissolved in 1 M diethanolamine pH 9.8, 0.5 M MgCl_2_. Absorbance was read at 405 nm after 1 hour at 37°C and IgG concentration determined by reference to a standard curve prepared with murine IgG (Statens Serum Institute).

### Western blot analysis

Western blotting was performed on leaf extracts as described previously [27].

### Statistical analysis

Statistical analysis was performed using GraphPad Prism 5 (GraphPad, San Diego, CA, USA). The differences in antibody titres from mice immunised with transgenic or wildtype plant extracts were analysed using a student’s T-test. Survival data were analysed by the Kaplan-Meier survival curve analysis.

## Supporting Information

Figure S1 **Confirmation of the stable transformation of tobacco plants with the** ***cps3S*** **gene.** Plant RNA was used as a template for reverse transcriptase PCR (RT-PCR) (Lane 1: wild type plants; Lane 2: transformed plants.) using *cps3S*-specific primers. RT-PCR products were analysed by agarose gel electrophoresis. The results show the presence of the *cps3S* gene in the transformed plants (Lane 2) but not the wild type plants.
(DOCX)

## References

[1] Gilbert C, Robinson K, LePage RWE, Wells J (2000) Heterologous expression of an immunogenic pneumococcal type 3 capsular polysaccharide in *Lactococcus lactis* . Infect Immun 68: 3251–3260. 10816470 10.1128/iai.68.6.3251-3260.2000 PMC97573

[2] Deuren M, Brandtzaeg P, van der Meer JW (2000) Update on meningococcal disease with emphasis on pathogenesis and clinical management. Clin Microbiol Rev 13: 144–166. 10627495 10.1128/cmr.13.1.144-166.2000 PMC88937

[3] CDC (2005) Direct and indirect effects of routine vaccination of children with 7-valent pneumococcal conjugate vaccine on incidence of invasive pneumococcal disease—United States, 1998–2003. 54: 893–897. 16163262

[4] Kunze R, Frommer WB, Flugge UI (2002) Metabolic engineering of plants: the role of membrane transport. Metab Eng 4: 57–66. 11800575 10.1006/mben.2001.0207

[5] Gao Y, Ma Y, Li M, Cheng T, Li SW, et al (2003) Oral immunization of animals with transgenic cherry tomatillo expressing HBsAg. World J Gastroenterol 9: 996–1002. 12717845 10.3748/wjg.v9.i5.996 PMC4611412

[6] Mason HS, Arntzen CJ (1995) Transgenic plants as vaccine production systems. Trends Biotechnol 13: 388–392. 7546570 10.1016/S0167-7799(00)88986-6

[7] Rigano MM, Alvarez ML, Pinkhasov J, Jin Y, Sala F, et al (2004) Production of a fusion protein consisting of the enterotoxigenic Escherichia coli heat-labile toxin B subunit and a tuberculosis antigen in *Arabidopsis thaliana*. . Plant Cell Rep. 22: 502–508. 14551732 10.1007/s00299-003-0718-2

[8] Arrecubieta C, Garcia E, Lopez R (1996) Demonstration of UDP-glucose dehydrogenase activity in cell extracts of Escherichia coli expressing the pneumococcal cap3A gene required for the synthesis of type 3 capsular polysaccharide. J Bacteriol 178: 2971–2974. 8631689 10.1128/jb.178.10.2971-2974.1996 PMC178036

[9] Jennings H (1983) Capsular polysaccharides as human vaccine. Adv Carbo Chem 41: 155–208. 10.1016/s0065-2318(08)60058-x 6195893

[10] Sharples SC, Fry SC (2007) Radioisotope ratios discriminate between competing pathways of cell wall polysaccharide and RNA biosynthesis in living plant cells. . Plant J. 52: 252–262. 17764499 10.1111/j.1365-313X.2007.03225.x

[11] Kalin M (1998) Pneumococcal serotypes and their clinical relevance. Thorax 53: 159–162. 9659348 10.1136/thx.53.3.159 PMC1745174

[12] Douglas RM, Paton JC, Duncan SJ, Hansman DJ (1983) Antibody response to pneumococcal vaccination in children younger than five years of age. . J. Infect. Dis. 148: 131–137. 6886479 10.1093/infdis/148.1.131

[13] Imöhl M, Reinert RR, van der Linden M (2010) Temporal variations among Invasive pneumococcal disease serotypes in children and adults in Germany (1992–2008). Internat J Microbiol 2010: 1–15. 10.1155/2010/874189 PMC2910462 20671944

[14] Snaidack DH, Schwartz B, Lipman H, Bogaets J, Butler JC, et al (1995) Potential interventions for the prevention of childhood pneumonia: geographic and temporal differences in serotype and serogroup distribution of sterile site pneumococcal isolates from children. . Pediatr. Infect. Dis. J. 14: 503–509. 7667055

[15] Hausdorff WP, Bryant J, Paradiso PR, Siber GR (2000) Which pneumococcal serotypes cause the most invasive disease: implications for conjugate vaccine formulation and use. Clin Infect Dis 30: 100–121. 10619740 10.1086/313608

[16] Levine MM, Levine OS (1997) Influence of disease burden, public perception and other factors on new vaccine development, implementation and continued use. Lancet 350: 1386–1392. 9365466 10.1016/S0140-6736(97)03253-4

[17] Hajdukiewicz P, Svab Z, Maliga P (1994) The small, versatile pPZP family of Agrobacterium binary vectors for plant transformation. Plant Molec Biol 25: 989–994. 7919218 10.1007/BF00014672

[18] Lund P, Dunsmuir P (1992) A plant signal sequence enhances the secretion of bacterial ChiA in transgenic tobacco. Plant Mol Biol 18: 47–53. 1731977 10.1007/BF00018455

[19] Draper J, Scott R, Armitage P, Walden R (1988) Plant genetic transformation and gene expression: a laboratory manual. Oxford, Blackwell Scientific Publications.

[20] Sawada H, Ieki H, Matsuda I (1995) PCR detection of Ti and Ri plasmids from phytopathogenic Agrobacterium strains. Appl Environ Microbiol 61: 828–831. 7574623 10.1128/aem.61.2.828-831.1995 PMC167346

[21] Fry SC (1988). The Growing Plant Cell Wall: Chemical and Metabolic Analysis. New York, John Wiley and Sons.

[22] Ouchterlony O, Nilsson,L (1973) Immunodiffusion and immunoelectrophoresis. Oxford, Blackwell Scientific Publications.

[23] Fry SC (2011). High-voltage paper electrophoresis (HVPE) of cell-wall building blocks and their metabolic precursors (Springer, New York, High-voltage paper electrophoresis (HVPE) of cell-wall building blocks and their metabolic precursors. The Plant Cell Wall Methods and Protocols. Z. Popper. New York, Springer: 55–80. 10.1007/978-1-61779-008-9_4 21222076

[24] Smith CK, Hewage CM, Fry SC, Sadler IH (1999) ±-d-Mannopyranosyl-(1->4)-±-d-glucuronopyranosyl-(1->2)-myo-inositol, a new and unusual oligosaccharide from cultured rose cells. Phytochem 52: 387–396. 10.1007/s004250050664 10592043

[25] Parsons HT, Yasmin T, Fry SC (2011) Alternative pathways of dehydroascorbic acid degradation in vitro and in plant cell cultures: novel insights into vitamin C catabolism. Biochem J 440: 375–383. 21846329 10.1042/BJ20110939

[26] Morton DB, Griffiths PH (1985) Guidelines on the recognition of pain, distress and discomfortin experimental animals and an hypothesis for assessment Vet Record. 116: 431–436. 10.1136/vr.116.16.431 3923690

[27] McNulty C, Thompson J, Barrett B, Lord L, Andersen C, et al (2006) The cell surface expression of group 2 capsular polysaccharides in *Escherichia coli*: the role of KpsD, RhsA and a multi-protein complex at the pole of the cell. Mol. . Microbiol. 59: 907–922. 10.1111/j.1365-2958.2005.05010.x 16420360

