# Supplementary material for: Recombinant Plants Provide a New Approach to the Production of Bacterial Polysaccharide for Vaccines
Source: PLoS One. 2014 Feb 3;9(2):e88144. doi: 10.1371/journal.pone.0088144 (PMC3912152; doi:10.1371/journal.pone.0088144)
Supplement: Figure S1 — Confirmation of the stable transformation of tobacco plants with the cps3S gene. Plant RNA was used as a template for reverse transcriptase PCR (RT-PCR) (Lane 1: wild type plants; Lane 2: transformed plants.) using cps3S-specific primers. RT-PCR products were analysed by agarose gel electrophoresis. The results show the presence of the cps3S gene in the transformed plants (Lane 2) but not the wild type plants. (DOCX) [file pone.0088144.s001.docx]

**SUPPLEMENTARY MATERIAL**

**Supplementary Figure 1. Confirmation of the stable transformation of tobacco plants with the *cps3S* gene.** Plant RNA was used as a template for reverse transcriptase PCR (RT-PCR) (Lane 1: wild type plants; Lane 2: transformed plants.) using *cps3S*-specific primers. RT-PCR products were analysed by agarose gel electrophoresis. The results show the presence of the *cps3S* gene in the transformed plants (Lane 2) but not the wild type plants.

**References**
